# Alternating evolutionary pressure in a genetic algorithm facilitates protein model selection

**DOI:** 10.1186/1472-6807-8-34

**Published:** 2008-08-01

**Authors:** Marc N Offman, Alexander L Tournier, Paul A Bates

**Affiliations:** 1 Biomolecular Modelling Laboratory, Cancer Research UK London Research Institute, Lincoln's Inn Fields Laboratories, London, WC2A 3PX, UK

## Abstract

**Background:**

Automatic protein modelling pipelines are becoming ever more accurate; this has come hand in hand with an increasingly complicated interplay between all components involved. Nevertheless, there are still potential improvements to be made in template selection, refinement and protein model selection.

**Results:**

In the context of an automatic modelling pipeline, we analysed each step separately, revealing several non-intuitive trends and explored a new strategy for protein conformation sampling using Genetic Algorithms (GA). We apply the concept of alternating evolutionary pressure (AEP), i.e. intermediate rounds within the GA runs where unrestrained, linear growth of the model populations is allowed.

**Conclusion:**

This approach improves the overall performance of the GA by allowing models to overcome local energy barriers. AEP enabled the selection of the best models in 40% of all targets; compared to 25% for a normal GA.

## Background

Impressive progress in protein structure modelling has been achieved over the last decade; however, improvement between subsequent rounds of the Critical Assessment of Techniques for Protein Structure Prediction (CASP) is often considered to be modest [1,2]. Given the current accuracy, protein models are useful for qualitative analysis and decision-making in support of a wide range of experimental work. High accuracy modelling is essential for important applications such as, molecular replacement experiments [3-5], function predictions [6] and virtual drug screening [7]. Modelling techniques, however, are still not accurate enough to close the gap between known protein sequences (approximately 5 million non redundant) and solved protein structures (approximately 50,000).

Regardless of the current limitations of modelling, two very encouraging observations have been made from the CASP7 results [1,8,9]. First, the gap between the quality of fully automated and manual modelling techniques has narrowed and second, improvement beyond the best template is achieved more frequently.

Modern template-based modelling pipelines can be divided into a number of common steps. A typical pipeline starts with template identification and alignment construction. In the next step, models are built using single templates, multiple templates or template fragments. The resulting models are then often refined, and finally the models are ranked and the "best" model selected.

Template search and alignment algorithms are showing significant improvements in accuracy and are becoming increasingly efficient. Well established sequence alignment algorithms such as FASTA [10], BLAST [11] and PSI-BLAST [12] are often replaced, or enhanced by more sensitive algorithms. These sensitive algorithms are based on multiple sequence alignments, sequence profiles or Hidden Markov models and take other information such as secondary structure prediction into account [13-17]. The impact of better alignments on the final model's quality is substantial, as errors made at this stage are not likely to be recovered during the subsequent modelling process.

Once a single template or several templates have been selected and the alignments constructed, models can be built. It is common practice to search the conformational space in order to further refine the structures [1,18,19]. Several different approaches have been developed for this task, using conserved constraints [20], genetic algorithms [21-25], Monte Carlo sampling [26], Molecular Dynamics [27], principle components analysis [28] or a combination of techniques [29-33]. Previous studies have shown that techniques combining several approaches perform best, when the different steps are carefully balanced [9].

For quality control and to reduce computational costs, protein model ranking and filtering can be applied at almost any stage of a modelling pipeline. Energy functions or statistical potentials are used to select a final model and usually form an integral part of the refinement method itself. Given their importance, the ability to select the best model based on energy alone is still relatively poor [34]. Moreover, most energy scoring methods are optimised in the context of specific modelling approaches, and applying them in a different environment may produce less reliable results.

Model selection has become an important field of protein modelling, and a separate category has been introduced in the 4^th ^Critical Assessment of Fully Automated Structure Prediction (CAFASP4) named Model Quality Assessment Programs (MQAP). The importance of this field was further underlined by a category called quality assessment (QA) introduced in CASP7 [35].

Several independent methods have been established and widely used to differentiate between models of high and poor quality [36-42]. Two different approaches can be distinguished; MQAPs scoring models in the context of model ensembles and MQAPs scoring single models independently. However, most of the top ranking MQAPs are dependent on the information of model ensembles [35,38].

Despite all of the above efforts and improvement in protein model construction, ranking and selection, it is still not possible to consistently produce models of high quality. To further progress template-based modelling, it is necessary to carefully evaluate each step and minimise the accumulated errors. Here, we describe a hierarchical modelling approach (template-based modelling), where each step has been carefully evaluated, giving new insights into generating and selecting better models. A known limitation of Genetic Algorithm (GA) approaches in protein modelling is that models tend to end up in local minima, not exploring the conformational landscape enough to be able to find the global energy minima. As a way to alleviate this situation we have implemented the novel concept of Alternating Evolutionary Pressure (AEP) into our Genetic Algorithm (GA) search engine. In AEP intermediate rounds of unrestricted linear growth are introduced, enabling the models to overcome small energy barriers. The AEP approach is shown to promote greater sampling of the conformational landscape thereby enabling better structures to emerge, thus facilitating final model selection.

## Methods

**Figure 1:**
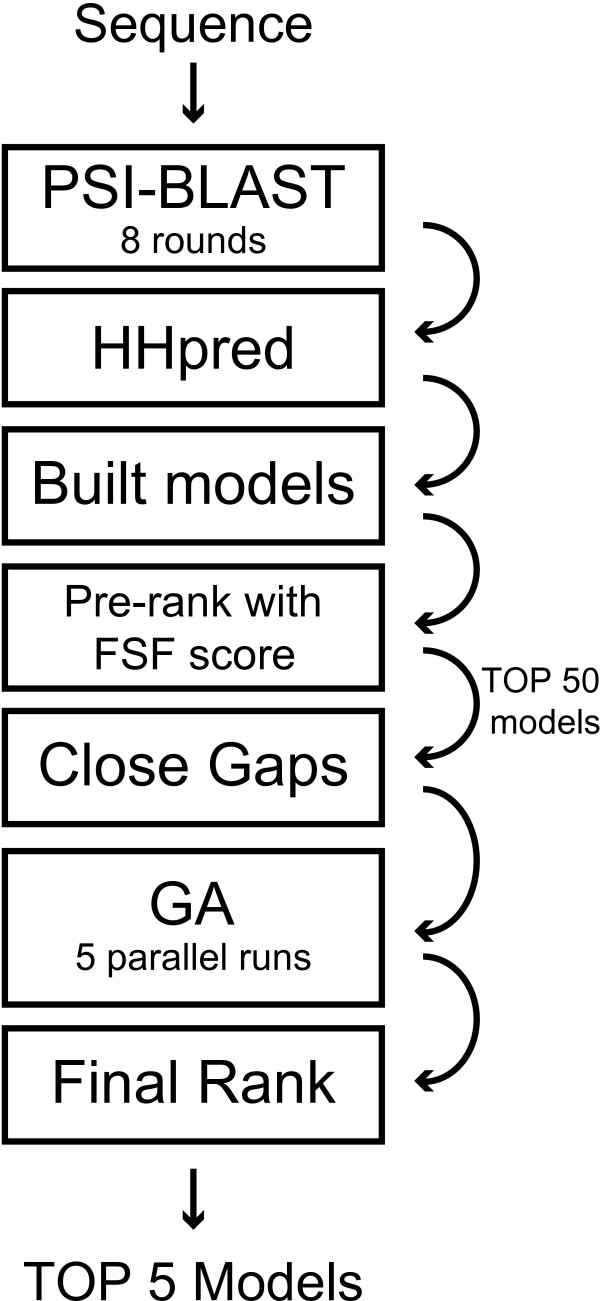
**Flow chart of modelling pipeline**. A sequence profile is created by performing eight rounds of PSI-BLAST. This profile is fed into the program HHpred to create a list of templates and alignments. All models are built by changing the amino acid sequence according to the alignment. The model ensemble is ranked using the FSF (see text for definition). The top 50 models are repaired, using the closing algorithm described in the text. These models are recombined in five parallel runs of our GA recombination with AEP (see text for definition). The created output models are ranked using DFIRE. The top five ranked models are returned.

In the following, the dataset, the algorithms applied and the pipeline of the modelling approach are described. The core of the method is an optimization protocol based on a Genetic Algorithm (GA). This approach mimics the principles of evolution, combining and mutating protein model ensembles. Details of the GA approach used to model and refine protein structures can be found in a previous publication [22]. To assure maximum yield, each step in the modelling pipeline has been evaluated separately. For an overview of the pipeline see Figure 1.

### Dataset

As the main objective of this work is to highlight problems in protein modelling and to extract potential solutions, the performance of the approach was benchmarked against well established modelling methods. We considered all sequences from the seventh round of CASP [43] which were downloaded from the Protein Structure Prediction Center webpage ([44]). The 104 protein sequences comprise 77 single and 27 multi-domain proteins. The final dataset consists of the 75 targets out of the 104 targets, for which reasonable templates could be identified.

### Template identification, sequence alignments and initial model building

Templates were identified and sequence alignments constructed using the Hidden Markov model based algorithm HHpred [15] in conjunction with PSI-BLAST [12] results and the pdb70 database, downloaded from the HHsearch webpage . Standard values were used for HHsearch, as provided by the software distribution. In order to allow a fair and unbiased comparison with all template-based CASP7 servers, the data set was restricted to the information available at the time of CASP7. Therefore, the PSI-BLAST results, PSIPRED [45] secondary structure predictions and selected templates were created using time-stamped data.

For the initial model building, all side chains were stripped off the templates, and the query sequence assigned to the backbone, according to the HHpred alignment. At this stage, neither insertions nor deletions were modelled or side chains added.

For several targets, we were not able to identify the substantially better templates used by the HHpred servers. This might be due to the fact that only the pdb70 database is available for download; the HHpred server uses a combination of the pdb70 and pdb90 database. It seems reasonable that better templates can only be found once both databases are used in conjunction, especially in cases where single, isolated, templates of higher quality are available.

### Repair algorithm

Due to deletions and insertions in the alignment, initial models are likely to be fractured. Missing residues within β-strands are especially hard to insert since it is very likely that any closing process will disrupt the precise hydrogen-bond network. On the other hand, substantial progress can be made focusing on coil and helical regions. We have developed a novel protein model repair protocol, which enables the modelling of incomplete coil and helical secondary structure elements with the correct length, thereby helping to further break away from the initial templates.

The new loop conformations are restricted to highly populated Φ/Ψ angles of the Ramachandran Plot [46] and backbone clashes are not allowed. The GA conformational search engine is applied to all initial models after backbone completion; it is, therefore, not necessary to extensively sample conformational space at this stage.

During processing, backbone bonds with non-standard length are first identified and fixed. For models with missing backbone elements, fragments are then created according to the PSIPRED secondary structure prediction, using internal coordinates with standard bond and angle values (IUPAC). These fragments are spliced into the backbone of the incomplete model and subsequently adjusted using a mechanism for closure, based on the cyclic coordinate descent algorithm [47]. The procedure is fully automated and only requires a protein model and the PSIPRED prediction. All parameters used in the closing algorithm's procedure were derived from our GA algorithm, which was trained on the CASP6 and CAFASP4 datasets. For algorithmic details [see Additional file 1].

### Model ranking

Several energy functions are used throughout the modelling pipeline. Preliminary investigations indicated that poor quality input models are not selected in the GA optimization process. Using model pre-ranking to remove these models at an early stage, allows more computational time to be spent on the better models. In the present approach, the best models are selected after optimization based on their energy scores. To quantify the ranking ability of our energy-scoring scheme for models, we calculate the Pearson correlation-coefficient between the energy and SC scores, defined below.

### Structure Comparison (SC) score

In order to assess the quality of the models generated, a measure describing the conformational similarity between models and the known native structure (target) was required. We used a structural comparison scoring scheme, defined as the mean of the scaled TM [48], GDT [49] and maxsub [50] scores. Since all three scores are scaled to the range [0, 1], the final SC score also ranges from 0 to 1.

$$\text{SC}=\frac{1}{3}\left(\text{TM}+\text{GDT}+\text{maxsub}\right)$$

### Energy scoring schemes

For each target all models were ranked using several different scoring schemes. A novel Fast Scoring Function (FSF) was used in the pre-ranking step. The FSF scoring scheme is composed of the following terms:

*S*_*FSF *_= *w*_1_*S*_*pp *_+ *w*_2_*S*_*cl *_+ *w*_3_*S*_*ss*_

Where *S*_*pp*_, the *residue-residue pair potentials*, is a score of the internal packing according to an empirically derived mixed backbone atom-centroid potential, as described previously [21,22]. *S*_*cl*_, the *clash penalty*, clashes are counted between two residues if any two backbone-atoms from any pair of non-consecutive residues are closer than 2 Å. *S*_*ss*_, the *secondary structure score*, is a sum of PSIPRED confidence scores for matches between predicted [45] and assigned secondary structure. The weights for the FSF were selected using the simplex algorithm [51] on the CASP6 and CAFASP4 datasets and are given by: *w*_1 _= 1.0, *w*_2 _= 2.4 and *w*_2 _= -2.4.

For scoring of the model populations during the GA optimization, the coarse energy score is used to preselect the models and reduce the population size to 100 models. Subsequently the fine energy score is used to further reduce the population to 50 models that are used for the next round.

The coarse scoring scheme includes the following terms:

*S*_*coarse *_= *w*_1_*S*_*pp *_+ *w*_2_*S*_*cl *_+ *w*_3_*S*_*ss *_+ *w*_4_*S*_*hb *_+ *w*_3_Δ*S*_*comp*_

Where *S*_*pp*_, *S*_*cl *_and *S*_*ss *_are defined as for the FSF. S_*hb*_, the number of hydrogen bonds calculated using the software STRIDE [52]. Δ*S*_*comp *_is the compactness reference score [22]. The weights for the coarse scoring scheme are: *w*_1 _= 1.0, *w*_2 _= 2.07, *w*_3 _= -4.20, *w*_4 _= -0.46 and *w*_5 _= 1.37.

The fine scoring scheme includes the following terms:

*S*_*fine *_= *w*_1_*S*_*eef *_+ *w*_2_*S*_*se *_+ *w*_3_Δ*S*_*comp*_

Where *S*_*eef *_is calculated in the following way. SCWRL 3.0 is used to replace all the side chains [53] for the energy calculation (standard parameters given by the program are used). All scored models are minimised and then scored using the effective energy function [54] (EEF) in CHARMM. *S*_*se*_, the solvent accessibility is calculated using the software POPS_A [55] and the solvation free energy [36]. Δ*S*_*comp*_, as previously defined [22]. The weights for the fine scoring scheme are: *w*1 = 1.0, *w*_2 _= 0.20 and *w*_3 _= 0.20.

After optimization with the GA protocol, the final models are ranked with a combination of the fine and coarse energy scores, and an all atom pair-potential score, DFIRE [56]. In the combined energy function the coarse and fine energy components are weighted according to the best template's sequence identity. Sequence identities were binned into three ranges: 0 – 0.3, 0.3 – 0.5, 0.5 – 1.0. For each of these ranges, the weights were optimised using the simplex algorithm on the CASP6 dataset, see Table 1.

**Table 1:** Weights for combined energy score

**Sequence identity**	**Weight coarse**	**Weight fine**
0 – 0.3	1	1
0.3 – 0.5	0.75	1
0.5 – 1	0.375	1

Weights for the final combined energy score assigned using the simplex algorithm, calculated from the CASP6 and CAFASP4 datasets. The higher the sequence identity, the less the coarse score is weighted.

The most representative structure of the final ensemble, which is taken from the middle of the largest cluster, was used as a control. This was done to investigate how consistently the top ranked solutions are selected, compared to the most representative conformation of the final ensemble.

### Energy minimization

To investigate whether full Cartesian space minimization facilitates protein model selection, all models, repaired and un-repaired, were minimised. The steepest descent and adopted basis Newton-Raphson methods were applied, as implemented in CHARMM [57] until the value of the gradient dropped below 1.0 kcal mol^-1 ^Å ^-2^.

### GA recombination

We previously described an efficient move-set used to search conformational space of protein models [22]. This move-set includes three global operators: the single and double crossover and the protein mutation operator; and two local operators: the helix and the coil mutation operators. A quick protein health check is performed during and after the application of the operators: Φ/Ψ angles must lie within the highly populated areas of the Ramachandran Plot and the change in energy-score is subject to a pseudo Metropolis criterion. The protocol was optimised using the CASP6 and CAFASP4 datasets.

The following modifications have been made to the protocol. Firstly, the input models are clustered using the nearest neighbour method. The metric for this clustering approach is based upon overall protein model similarity weighted with the secondary structure scores. Only the largest two clusters are used for further optimization, thereby removing a few, poor outliers. Secondly, the range of movement for mutations has been changed, to allow finer movements. This was achieved by allowing all values within the highly populated areas of the Ramachandran plot.

After applying the closing algorithm to all selected models, the models were submitted to five parallel runs of the GA protocol. The optimization is run for at least five, and a maximum of 10 rounds, dependant on the population convergence. Running the GA for longer was found to increase the probability of ending in incorrect local minima conformations (data not shown).

### Alternating evolutionary pressure

In a GA where Alternating Evolutionary Pressure (AEP) is applied, a number of non-scored rounds are allowed between each scored sampling round. In these non-scored rounds, the population grows linearly and the structures in the ensemble are allowed to sample energetically unfavourable states. Although energy evaluation is not applied, to ensure reasonable sampling, basic protein health checks associated with the operators are still in place. Four different setups were applied: a normal GA and a GA with one to three non-scored intermediate rounds. In each setup, 10 fully ranked rounds were performed where the population was reduced to the top 50 models.

### Accessibility

The complete modelling procedure can be accessed via a web server interface at: [58]. The average running time for a protein model of 150 residues is 6–7 hours for the standard GA and 15–25 hours if two intermediate non-scored rounds are used (AEP2). For details of this server [see Additional file 1].

## Results and discussion

For this study we modelled 75 diverse protein sequences from the CASP7 dataset of the category template-based modelling. First, a novel backbone repair algorithm is introduced and compared to the performance of MODELLER. In the next step an optimal setup for pre-ranking is investigated. The resulting models are recombined using a GA and the improvement in model selection due to the introduction of AEP is shown. Finally, a summary is given showing the performance of several possible modelling pipelines.

### Structural comparison of models before and after repair

We have developed a novel algorithm for completing and closing protein backbones. Coil and helical regions are completed and the length of incomplete helical secondary structure elements is adjusted to agree with the predicted secondary structure. In contrast to other loop modelling methods, only a single conformation is created for each added structural element. These conformations are further sampled once the model undergoes recombination using the GA.

**Figure 2:**
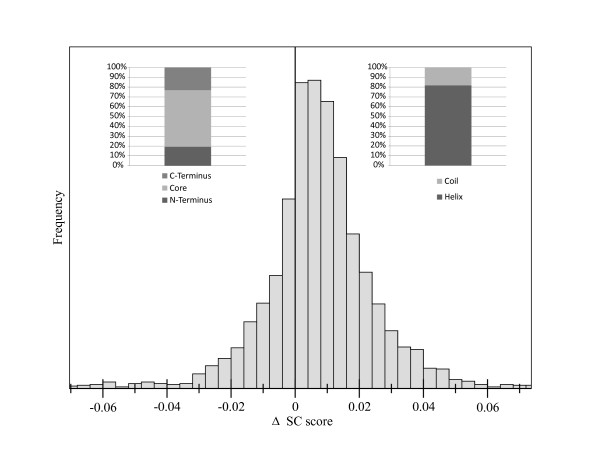
**Distribution of the ΔSC score after model repair**. The frequency of ΔSC scores for repaired models is shown. Models with negative values have a decreased SC score after backbone repair. Improved models have a positive value. The distribution is shifted towards model improvement. In the insets, it can be seen that more than 80% of improvement lies within helical regions. Most of the improvement is situated in the protein core, the region between the two terminal elements.

The distribution of improvement in SC score due to this repair process is shown in Figure 2. In 72% of all cases, completed structures show improvement in comparison to their initial score. The models improved by the repair algorithm show an average improvement of 0.015 SC score and the best 25% of the population (3^rd ^quantile) shows an improvement greater than 0.02 SC score.

Analysing the SC score in terms of the assigned secondary structure, we found that approximately 80% of the improvement made for all models lies within helical secondary structure elements (see Figure 2 inset). In contrast to this, only approximately 20% improvement is gained completing coil regions. Approximately 60% of the improvement is situated in the core region, defined here as the region between the N and C terminal secondary structure elements. The rest of the improvement is located within the termini.

Comparing the models derived from the top ten alignments of each target, with the equivalent models constructed with the automodel function in MODELLER [59], the repairing algorithm scores on average 0.428 SC score compared to 0.427 SC score for MODELLER. Although the score for the closing algorithm is not significantly better, this method allows repair of models without the need for the alignment and/or template.

### Initial ranking

For modelling pipelines with extensive conformational search algorithms it is not obvious when to rank models. Ranking can be applied at several stages, such as before insertions and deletions are dealt with, after backbone completion, after minimization or after refinement. Intuitively, one might think that backbone completion is a minimum criterion to be fulfilled before further consideration on model quality can be made. To address this question, we analysed the effectiveness of the FSF and DFIRE scoring schemes before and after repair.

**Figure 3:**
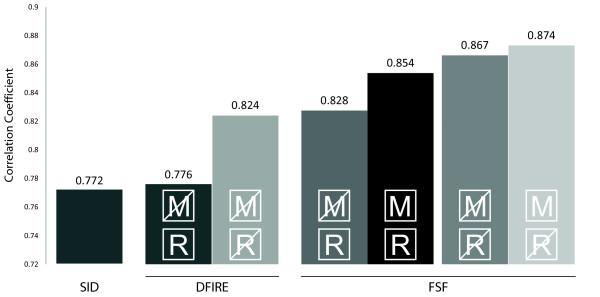
**Correlation-coefficient for FSF score and DFIRE**. The Pearson correlation coefficient is shown for different model datasets and scoring functions. Weighted SID, DFIRE and the FSF are used for the ranking. Repaired (R), non-repaired (crossed R), minimised (M) and non-minimised (crossed M) models are ranked and compared. Repairing models decreases the correlation coefficients for DFIRE and FSF. Minimisation further improves the ranking ability of the FSF. The best correlation-coefficient can be observed for minimised, non-repaired models.

Figure 3 presents the Pearson correlation coefficient, i.e. the correlation between SC score and energy score, for different setups, and scoring schemes (the Spearman correlation-coefficient shows similar results). Surprisingly, the ranking of repaired models produces a lower correlation-coefficient than the ranking of un-repaired models. The same trend is seen whether the FSF or the DFIRE energy function is applied, showing this effect to be independent of the actual energy-based/statistical scoring function used. This observation can be explained by the following. Models derived from alignments with fewer insertions and deletions tend to be closer to the template and are generally of better quality. Due to the use of pair potential energy functions, models with more residues tend to have better energies. Hence before repair, the better models with fewer insertions/deletions have lower energy scores. Once repaired, this effect disappears and the advantage gained from the better initial template quality is not picked up by the energy functions anymore.

On the other hand, ranking according to the alignments scores given by the alignments algorithms is normally not sufficient for model selection either. Ranking purely based on the coverage dependant sequence identities of the alignments, produced a correlation score of 0.772, 12% smaller than the best FSF ranking.

In Figure 3 it can be seen that the best ranking is obtained using the FSF on unrepaired, minimised structures, improving the correlation by 6% compared to the best DFIRE configuration and by 13% compared to ranking using the weighted SID. Non-repaired models are generally easier to rank, this is valid both for DFIRE and the FSF. Ranking unrepaired models results in an improved correlation coefficient of 6.2% for DFIRE, 4.7% for the FSF. Minimization further facilitates ranking ability for unrepaired models by a further 1% using the FSF.

### Recombination

**Figure 4:**
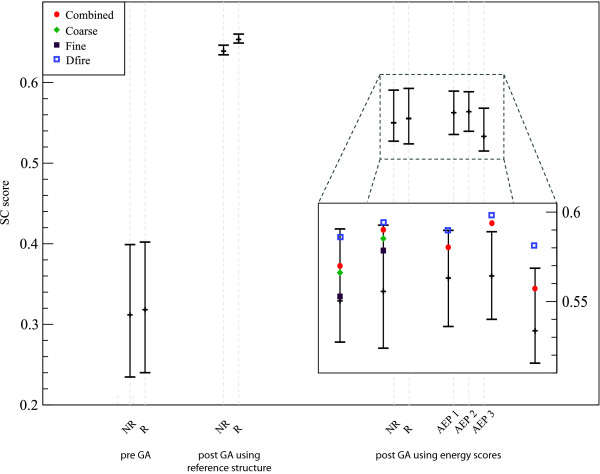
**Population distribution pre- and post-GA**. The median, first and third quantile are shown for three different setups. In each setup the average of all model distributions are given for repaired (R) and non-repaired (NR) models. The population for the initial, pre-GA models is broad and lies well below the distribution for the post-GA populations. Repaired models show only small improvement for the pre-GA and post-GA energy based model ensembles. However, a clear advantage can be seen once the GA which is directly driven towards the native structure is applied. The populations for the energy driven GA runs can be seen in more detailed in the graph inset. Here, it is also shown how well good models can be selected using different energy functions. These scores are for the averages of the SC scores for the lowest-energy model of each target. The energy functions used are the combined (red dot), the coarse (green diamond), the fine (purple filled square) and DFIRE (blue empty square) for the standard GA and AEP1-3. As the combined energy and DFIRE score has been found to produce the best results, the other scores are not shown for the AEP. The best model selection is seen for AEP2 which has a narrower population distribution with some good individual outliers. The distribution for AEP3 shows a drop in good models; furthermore, a decreased ability to select good models is shown.

To further sample the conformational space and select a good final model, all repaired models were recombined and optimised using the GA. Figure 4 shows the median, first and third quantile of the different modelling populations pre/post GA. All GA runs only have ten fine energy scored rounds. Applying more than ten fine energy rounds increases the probability of convergence of the model ensemble into a local minimum [see Additional file 1, Figure S3].

Running the GA optimization using the SC score to the native protein structure as the fitness function shows how much improvement can potentially be [see Additional file 1, Figure S2]. After the application of the GA using the native structure as guidance, the model ensemble is very narrow and on average the final population is improved by 51% to an SC score of 0.643 for non-repaired and 0.658 for repaired models. Interestingly even in this ideal scenario the move set is unable to produce better structure due to a lack of good quality templates, absence of secondary structure elements in the model population or insufficient sampling. It can also be seen that repaired models clearly improve the overall population, due to the models not missing secondary structure elements. Longer sampling further increases the improvement, but for the purpose of comparison we limited the sampling to 10 rounds.

In practice we do not have access to the reference structure and have to rely on energy scores to drive the GA. Figure 4 also shows the results using different energy scores for final model selection. It can be seen that repairing structures does improve the top model, for all energy functions used. The best results can be obtained using the DFIRE energy function producing a 2.4% improvement compared to the fine score, a 1.4% improvement compared to the FSF score and a 0.5% improvement compared to the combined energy function.

The difference between the average SC score for the best models created using the energy and SC score to drive the GA is only 7%. However, a further 4% improvement is lost when selecting the model with the lowest energy out of the final energy driven ensemble.

### Alternating evolutionary pressure (AEP)

**Figure 5:**
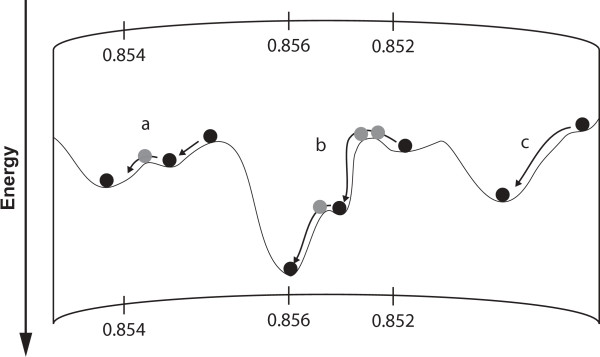
**Energy landscape**. Due to energy barriers, some models need to be nudged in order to fall into their optimal energy basins. A relatively small change in structural conformation can have a marked effect on a model's energy; SC scores are given along the energy landscape (top and bottom). A curved representation is chosen to highlight the three-dimensional nature of energy landscapes. Dark circles are conformations that are scored; light grey circles are non-scored, intermediate, conformations. In case (a) one non-scored intermediate conformation is needed to climb a small energy barrier. A more difficult case is shown in (b), where a maximum of two consecutive intermediate conformations are required, before energy evaluation. Case (c) shows a scenario where no intermediate conformation is required.

GAs and other similar conformational search algorithms suffer from the problem that they tend to stay within local minima instead of exploring further afield and potentially finding a deeper minimum. We investigated whether alternating evolutionary pressure (AEP) could facilitate energy based model selection, by gently pushing models over small energy barriers. This idea is illustrated in Figure 5, showing that small changes in protein structure, although insignificant in terms of the SC score, produce significantly better energies, hence facilitating protein model selection.

Two main elements dictate the success of GA approaches. One is the set of operators (move-set) and the other is the fitness function (energy scoring scheme). Classically, GAs operate for several generations, iteratively applying the conformational search engine and the fitness function [60] until convergence is obtained. However, interesting results can be observed, once a series of conformational changes are applied, without intermediate population scoring and reduction. Within these non-scored intermediate rounds the population grows linearly. The finer energy evaluation and the reduction of the model ensemble to the best members are not applied; however, during these rounds the basic protein health checks of the operators are still applied (see Methods).

A similar approach to AEP was introduced by Qian et al., where the refinement of protein structures was achieved using an iterative alternation of diversification and intensification steps [61]. This approach combines ideas from tabu search and conformational space annealing, however, it is only applied if the lowest energy refined structures have not converged and show several variable and less reliable regions. In general, this methodology is different from classical GAs where the optimization process is more variable, less directed and, therefore, convergence is only achieved after intensive sampling. For these classical GAs, the principle of AEP has only been used before to provide theoretical predictions of algorithm performance [62]. Here, we take this idea one step further by removing the ranking step for a number of intermediate rounds. We used four different setups: the standard GA and the GA with one, two and three non-scored intermediate rounds. The results presented in Figure 4 show that our ability to identify the better conformations varies strongly depending on the number of AEP rounds used. For each GA setup, we calculated the percentage of targets for which the best model based on SC score also had the lowest energy score. Using the standard GA protocol, the best model was identified in 25% of all targets using the DFIRE energy scores. Similar results are produced with a single (AEP1) intermediate round (31%). For the runs with two intermediate rounds (AEP2) the best model was identified in 40% of all targets. However, for three intermediate rounds (AEP3) the selectivity dropped to 30%.

Allowing two intermediate, un-scored GA rounds yield the best results for model selection in this analysis. In this setup, small energy barriers can be overcome, producing some very good individual models with low energies. However, once the evolutionary pressure is too low, as seen for the AEP with three intermediate rounds, the whole population drifts away and the quality of the lowest energy model decreases.

**Figure 6:**
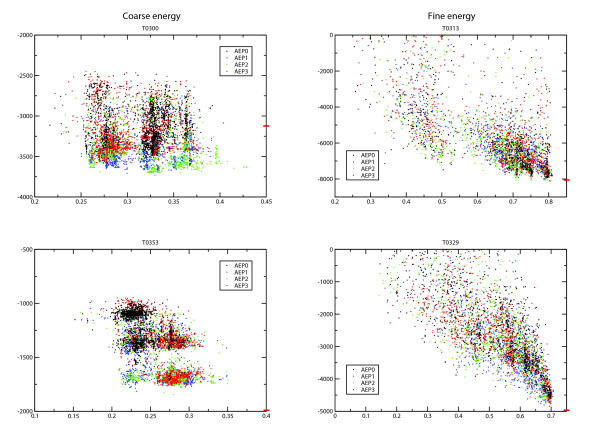
**Sampling with the normal GA and AEP**. The coarse and fine energy (y-axis) versus the SC score (x-axis) is plotted for 10 equally scored rounds, of four representative targets: T0300 (coarse), T0313 (fine), T0329 (fine) and T0353 (coarse). This is done to show the distribution of the models' coarse and fine energy scores; however, for all four cases both energy scores are used for ranking, as described in the methods. For the two coarse energy plots the corresponding energy funnel for the fine scoring scheme is also not particularly well defined, however, certain trends are easier to observe by plotting the coarse scoring scheme for these cases (see text). AEP0 (standard GA) is coloured black, AEP1 red, AEP2 green and AEP3 blue. For all four cases the energy of the native structure is shown as a red dash on the right y-axis of each graph. In the case of T0300 the energy of the native structure is much higher than a large proportion of the models. This can explained due to a particularly poor agreement between predicted and native secondary structure.

In Figure 6 we present the coarse energy distributions for two representative remote homology targets, T0300 and T0353, and fine energy distribution for two high homology targets, T0313 and T0329. Energies for the normal GA and AEP1-3 are shown for each distribution. Generally it can be seen that the energy funnel is less well defined for lower homology modelling, T0300 and T0353, a known observation for energy-based model ranking. The advantage of the optimization process with AEP2 (green) is illustrated in T0300. In this energy plot it can be seen that more sampling of higher quality models is found for AEP2 (green). Indeed, for 80% of all cases, including T0300, T0329 and T0353, AEP2 produced the best results in the final selection. T0313 and T0329 are examples where sampling with the standard GA is sufficient. In the cases of T0300, T0329 and T0353 the problem of local minima for AEP3 (blue) can be seen, where some individual models of poorer quality have very low energies compared to all other sampled models. Sampling of the normal GA is often not as thorough as for AEP, which can be seen in T0300 and especially well in T0353, two harder modelling targets. AEP1 is sampling more space than the standard GA, but still less than AEP2 and produces inferior results.

In order to further understand the effects of AEP we applied several "normal GA" runs with adapted parameters. First standard GAs were run with the same number of rounds as given for AEP1-3; these runs produced significantly inferior results compared to the standard GA. This effect can be explained by the oversampling of local minima, which could not be prevented even using statistically derived constraints (constraining the less variable structural regions). Additionally, we increased the population size for normal GAs to 500, 1000 and 1500 models per round, which increased the computational costs but did not show any improvement in model quality for the lowest energy ranked models. In general, it seems that the AEP2 protocol gives the better balance between sampling the variable regions without drifting too far away in regions that are more structurally conserved; models that undergo consecutive multiple mutations in the structurally conserved regions are less likely to survive.

Below, we present two cases; the first where there is no improvement using AEP; the second where significant improvement could be achieved having two intermediate, un-scored, sampling rounds.

#### Case I: T0380

For T0380 a β-strand mainly protein with 145 residues had to be modelled. With the template search 17 different templates were identified and, after backbone completion, the SC scores ranged from 0.292 – 0.761. Only one high quality template was identified for the starting population. The best member in all final ensembles had a SC score of approximately 0.77. For none of the four recombination-setups were we able to select a model close to the best input. The worst results were created using the AEP3. All selected models ranged between a SC score of 0.609 to 0.632. Interestingly, recombination of non-repaired models enabled the selection of a final model with a score close to 0.772. After backbone completion, the energy function was not able to distinguish between a good model derived from the best template, and an inferior model, produced by the closing algorithm.

#### Case II: T0311

**Figure 7:**
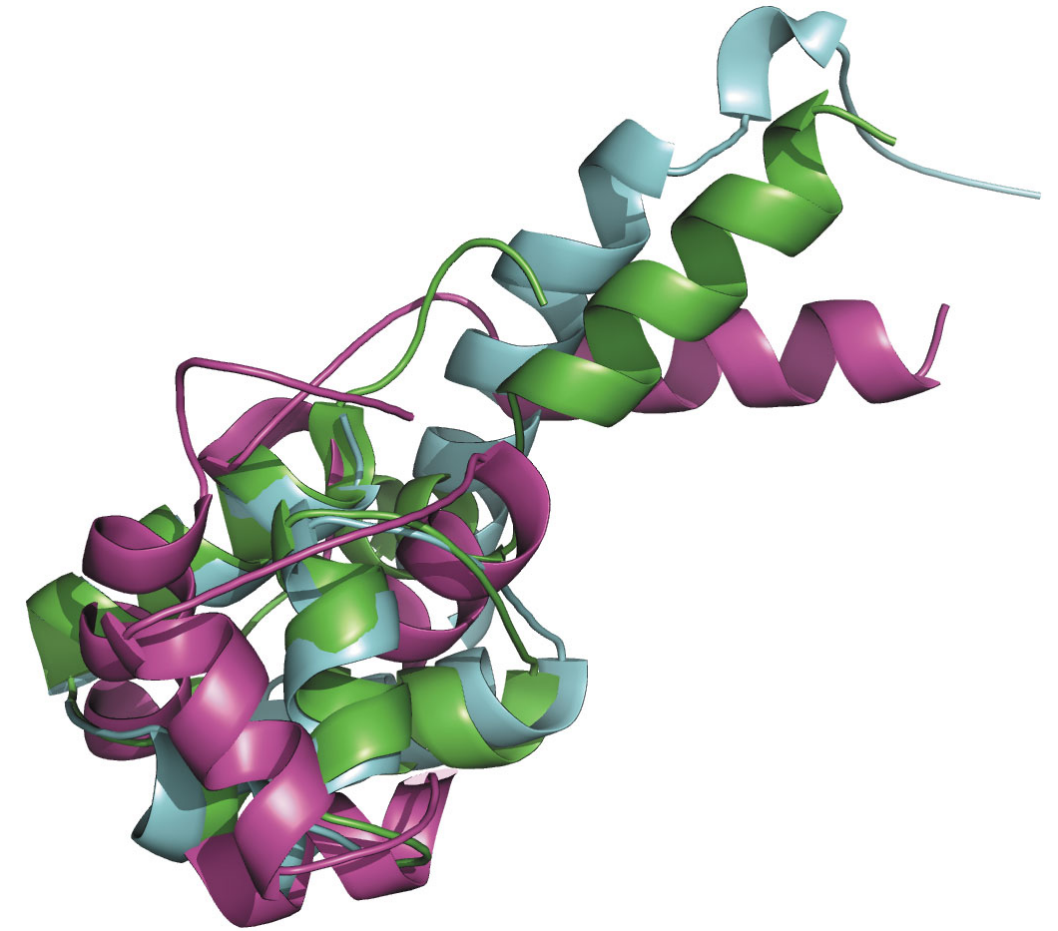
**Superimposition of models and target for T0311**. The lowest energy model for the standard GA run (purple) and the lowest energy model for the GA with alternating evolutionary pressure (green) are superimposed onto the native structure (light blue). It can be clearly seen that the majority of helical elements superimpose better on the model produced with alternating pressure, resulting in a backbone RMSD of 3.13 Å. The model produced with the standard GA has a backbone RMSD of 7.81 Å to the native structure.

In this case an all-helical protein with the length of 88 residues has been modelled. The sequence search produced a list of 165 potential templates. The top ranking alignment produced a model with a SC score of 0.600. The best, repaired, input model for the recombination had a SC score of 0.617. Applying the standard GA selected a model of relatively poor quality with a SC score of 0.575. However, allowing two non-scored intermediate rounds improved the model beyond all initial input models and aided selecting the best member of the final ensemble, based on energy. For this final model, which has a SC score of 0.637, two helical secondary structure elements show improved positioning relative to the native structure. The native structure and the best models for the standard GA and the two intermediate rounds GA are shown superimposed in Figure 7. Clearly, the overall topology of the non-standard GA (backbone RMSD 3.13 Å) is improved relative to the standard GA model (backbone RMSD 7.81 Å), having several helical elements in the correct orientation.

### General improvement along the modelling pipeline

**Figure 8:**
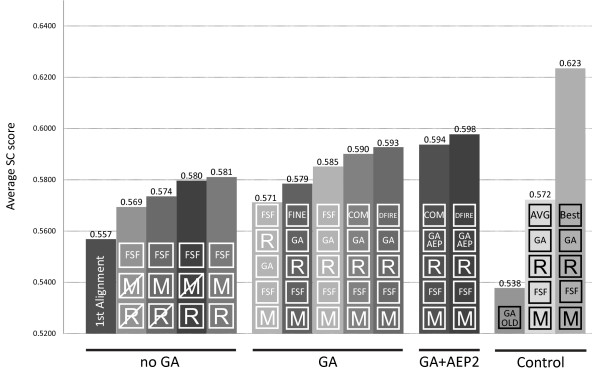
**Summary of average top ranks**. Final results produced by different pipeline setups are shown. The flow of each setup is defined by symbols, ordered from bottom to top. The following symbols are given: R – repair, M – minimise, FSF – selection with FSF, FINE – selection with the fine energy function, COM – model selection with the combined energy score, DFIRE – selection using the DFIRE energy function, AVG – selecting the centre of the clustered population, BEST – selecting the best model, GA – application of the GA, GA AEP – application of the GA using alternating evolutionary pressure and GA OLD – application of the previously described GA protocol. The best pipeline uses the AEP2 GA for sampling and the final models are selected using DFIRE.

The results for the different pre- and post-GA conditions are compared in Figure 8. Here we compare several possible modelling pipelines. The first pipeline considered, consisted of model selection without application of the GA. In this context the best final models were obtained using the FSF on minimised and repaired models. This result seems to contradict our observations on ranking correlation; however, here the emphasis is on final model selection without further refinement of the population. For this setup, an improvement of 4.1% can be seen compared to the models derived by the initial alignment.

For all pipelines using the GA with or without AEP, the unrepaired models were pre-ranked and the resulting model population was repaired before optimization. Since it was shown that un-repaired models are easier to rank, one might think that backbone completion should be the final step after recombination. To test this we compared both possibilities and it can be seen that performing backbone completion before the GA rather than after provides an improvement of 1.4% SC score; this can be explained by the additional sampling of the added secondary structure elements in the GA optimization.

The performances of the fine, the FSF, the combined and DFIRE energy score are compared for final structure selection. The best results for the energy-driven GA were obtained using DFIRE which improves the average SC score by 2.4% compared to the fine, 1.4% compared to the FSF and 0.5% compared to the combined energy score. Using DFIRE for the final model selection improved the average SC score for the normal GA from 0.590 to 0.593.

Use of AEP2 during recombination further improved the final models' quality from 0.593 to 0.598 in SC score using DFIRE for final selection. DFIRE also performs better than the combined energy score for the final selection in AEP2. This shows the importance of using a final model selection scoring function that is not used for the optimization procedure.

Overall, an improvement of 6.8% is achieved for the optimum modelling pipeline (AEP2 + DFIRE) compared to the models derived from the first alignments. Comparing these results to the SC scores of all models produced by automatic servers during CASP7 for our 75 targets, the normal GA would rank 12^th ^and the AEP2 5^th^.

Consistent selection of the best SC score model would enable a further improvement of up to 4%. Clustering, as described above, was used as an alternative selection protocol to identify the most representative models, but produced inferior results; on average 4.3% lower than our optimal setup. Visual inspection of the final model ensembles indicated that the better structures are often isolated from the largest clusters.

Overall, in 77.3% of all targets, the best model of the final ensemble had a greater or equal SC score compared to the best model of the initial input population. A similar trend was observed for the lowest energy models, where 69.3% of the lowest energy model of the final population had greater or equal SC score compared to the lowest energy model of the initial population. For 18.7% of all cases the final lowest energy model was improved in SC score than the best model of the initial input population, thereby selecting or improving the best model.

**Figure 9:**
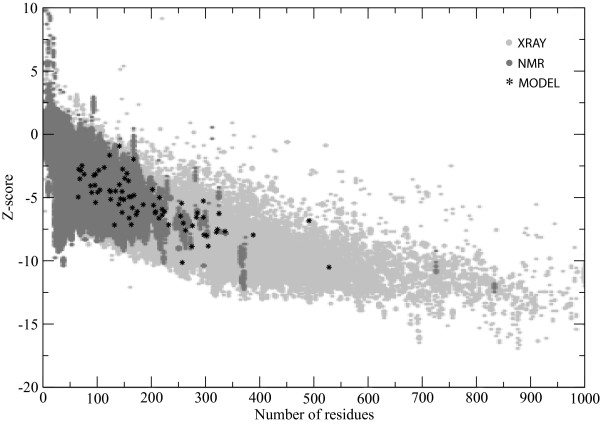
**ProSA scoring of top models**. All top models, selected with DFIRE, were checked using the ProSA web server. Here, the z-scores are plotted against the protein size. Models are shown as black stars. As a control, the distribution of X-ray (light) and NMR (dark) structures from the RCSB Protein Data Bank are shown. This plot has been adapted from the results of the ProSA web server [63].

Finally, possible structural errors in the selected models were investigated, using the ProSA web server [63] which compares them to X-ray crystal and NMR structures. As can be seen in Figure 9, all 75 models produced energy-related z-scores comparable to the scores of X-ray crystal structures. Furthermore, it has been shown for our high accuracy CASP7 submissions that we ranked 8^th ^of all submitting groups for the accurate prediction of the χ1/χ2 angles [19]. The move set of the GA has not been changed for this work, and therefore these findings remain valid, indicating that the conformational sampling performed in internal coordinate space does not adversely affect the side chain quality. However, as a further check of model quality, a subset of randomly selected models plus the final models selected with the best overall pipeline were also tested for stereo-chemical properties using the PROCHECK [64] software package. These models showed a quality comparable to the other top-ranking models submitted to CASP7.

## Conclusion

In the present work we have performed a detailed analysis of the different steps that form the pipeline of our template based GA protein modelling approach. The results shown here, clearly demonstrate that pre-ranking should be applied to unrepaired models before backbone completion. Ranking of these incomplete models using the novel FSF scoring scheme combined with minimization was shown to provide the best ranking (13% improvement compared to weighted sequence identity). This method could be used to pre-select a final model in protocols where rapid modelling from single templates is necessary.

DFIRE was found to be the most efficient way to select the top model (0.593 SC score). The selectivity of DFIRE can be further improved by introducing Alternative Evolutionary Pressure (AEP) to the GA protocol (0.598 SC score). Creating subtle movements in the protein models using AEP, helps to select the better models by nudging them to lower energy states.

When using GAs for protein modelling two different effects can be achieved. First, GAs can be applied to improve models beyond the best input structure. Second, GAs can be used to ease the selection of the better protein models by lowering their energies. However, lowering the model's energy does not necessarily improve the structural score. In the approach used here, the GA was used to improve selection of good models. Improvement beyond or maintaining the best input model was seen for 77.3% of all targets. However, these models could only be identified in 25% for the normal GA, 31%, 40%, 30% for AEP with 1–3 intermediate rounds respectively.

The application of all the above strategies improves the final average structural score by 7.4% compared to the purely alignment-based pipeline. This was achieved by a carefully balance between the number of sampled intermediate structures (AEP2), the scoring functions used in the GA and the final selection of models with DFIRE. Overall, this pipeline would rank 5^th^, comparing these results to the scores of all models produced by automatic servers during CASP7 for our 75 targets

Further investigations and development need to be undertaken in order to make full use of the GA/AEP conformational search engine. Other techniques will undoubtedly be required to further assist conformational search engines, such as GAs, to recover from local minima. In general more work is required to refine scoring schemes so that the best models can be consistently selected from the ensembles of structures.

## Competing interests

The authors declare that they have no competing interests.

## Authors' contributions

MNO and PAB devised the work. MNO carried out all computational work and wrote the initial manuscript draft. ALT and PAB edited the paper. All authors read and approved the final manuscript.

## Supplementary Material

Additional file 1 Supplementary material. This file provides a detailed description of the protein backbone repairing algorithm, detailed results of the GA recombination for structure and energy-guided optimizations and information on the 3D-Jigsaw 3.0 protein modelling web-server. Click here for file
