# Supplementary material for: Alternating evolutionary pressure in a genetic algorithm facilitates protein model selection
Source: BMC Struct Biol. 2008 Aug 1;8:34. doi: 10.1186/1472-6807-8-34 (PMC2527322; doi:10.1186/1472-6807-8-34)
Supplement: Additional file 1 — Supplementary material. This file provides a detailed description of the protein backbone repairing algorithm, detailed results of the GA recombination for structure and energy-guided optimizations and information on the 3D-Jigsaw 3.0 protein modelling web-server. [file 1472-6807-8-34-S1.pdf]

Supplementary material for:

Alternating evolutionary pressure in a genetic algorithm facilitates protein model selection

Marc N. Offman, Alexander L. Tournier and Paul A. Bates

Table of Contents

Repair algorithm **1**
Identification of breaks 2
Closing geometrical breaks 2
Modelling of missing parts 3
Recombination **5**
Server **6**
Bibliography **10**

Repair algorithm

In the repair algorithm, first, backbone-bonds with non-standard-length are identified and fixed. Second, fragments are created according to the secondary structure prediction, using internal coordinates with standard bond and angle values (IUPAC). These fragments are spliced into the backbone of the incomplete model and subsequently adjusted using an algorithm based on the cyclic coordinate descent (CCD) algorithm[1]. In this algorithm the Ramachandran Plot[2,3] is used for Φ/Ψ sampling, clashes are prevented and the convergence criterion is based upon structural similarity between the initial and repaired fragment stems. The exact procedure is outlined below.

Identification of breaks

Various categories of breaks can appear in protein models. Here we broadly distinguish between breaks, arising from incorrect peptide bond lengths or deletions from the template (geometrical breaks, II) and those caused by missing residues (sequence breaks, I & III) (Figure S1 a). Geometrical breaks were identified measuring the peptide bond length between N and C atoms of adjacent residues. Missing residues were identified using the residue numbering, and the single sequence alignment[4] between the full query and the model sequence.

The probability of introducing errors to a protein structure increases with the length of missing secondary structure elements to be inserted. Longer insertions often hinder the completion of subsequent breaks. Thus, it is advisable to define an explicit order and maximum length for insertions. This length is set to 15 residues for coil regions and 25 for helical regions. Moreover, we decided to complete the backbone in the order from short to long fragments.

Closing geometrical breaks

If a geometrical break is within 10 residues of the terminal regions, the whole secondary structure element is remodelled; otherwise the CCD algorithm is applied. In the initial attempt, flanking residues on either side of the break are included to facilitate closure. If this attempt fails, the number of surrounding residues is expanded stepwise. However, this expansion is not allowed to include residues outside the coil element that contains the break. When a break lies within a helical structure, the whole helix is replaced, remodelled with its correct length and fitted into the original model.

In some cases, a geometrical break is proximal to a sequence break. If these are less than five residues apart, they are realigned, thereby reducing the amount of conformational change needed to be dealt with (see Figure S1 b). Although this procedure results in a local realignment, it prevents major rearrangements of the backbone and prevents total failure of the closing algorithm.

Modelling of missing parts

Using PSIPRED[5] secondary structure prediction, broad three-state conformations for the missing secondary structure elements are assigned. Angles, distances and torsion angles comply with the official IUPAC definitions. All residues, within a modelled insertion, have torsion angles distributed within the highly populated areas of the Ramachandran Plot. These elements are modelled separately and spliced into the initial model. After the insertion of the new secondary structure elements, the resulting breaks in the protein backbone are closed using CCD algorithm, sampling highly populated Φ/Ψ angle combinations.

If a sequence break is proximal to either terminus, the CCD algorithm is not required and new residues are simply added to the model. The conformation of the addition is checked for backbone clashes, using the coarse backbone clash score[6]. If backbone clashes occur, the terminus is remodelled.

If a sequence break is not proximal to either terminus, a method to maintain the overall topology is required. The CCD algorithm is applied, and a closed conformation is accepted, once the SC score between the closed and initial structure is above 0.98.

Easier cases are coil only insertion, where either a full loop or only a few residues of a loop are rebuilt. The missing residues are spliced in and if the loop does not close initially, the flanking residues around the break are extended stepwise, up to five residues aside. However, the extended flanks need to be part of the same loop.

Insertions of mixed secondary structure elements are more complicated and only the coil residues of these elements are used during the CCD closure. If it is not possible to close the gap using these residues, the flanking regions around the gap are also extended. In addition, small adjustments are allowed to the Φ/Ψ torsion angles of helices and strands. If an incomplete helix is between two coil elements, the whole helix is removed and replaced by a new, completed one. The flanking coil-regions are adapted to allow closure.

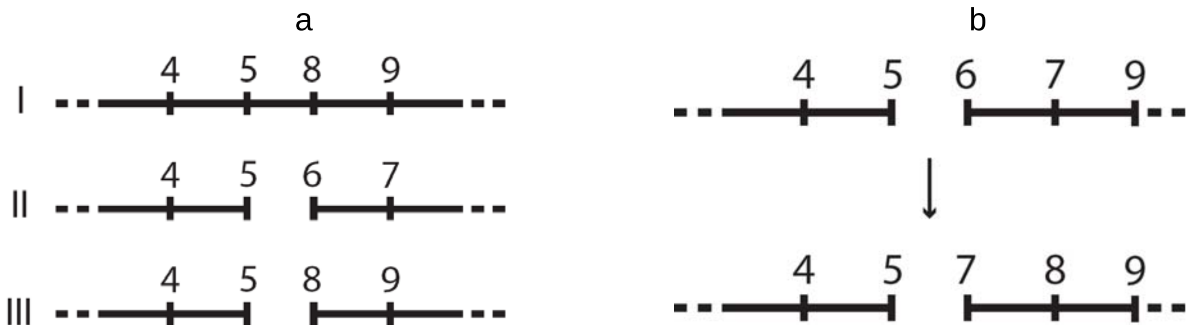

Figure S1 Different types of backbone breaks.

a) In case I the backbone of the structure is closed, two residues are missing and a “bond” exists between two, non-adjacent, residues. Case II shows a break in the model, derived by a deletion in the alignment. Case III shows a break in the backbone with missing residues.

b) A geometrical break between residues five and six can be seen. Furthermore, residue eight is missing. The model’s backbone is realigned, superimposing the geometrical break and the missing residue, creating only one gap to model.

Recombination

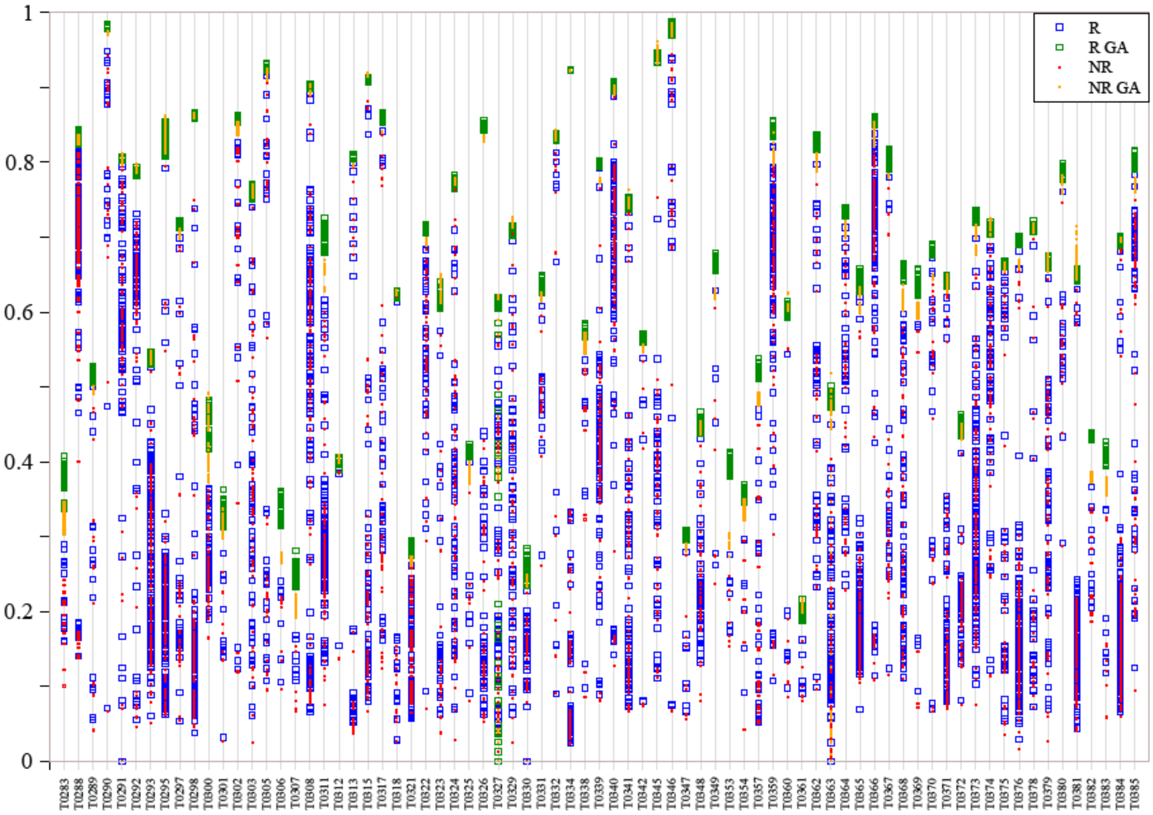

Figure S2 Structure driven GA runs.

For all 75 targets the input populations and final populations after refinement, are shown. The y-axis shows the SC score to the native protein structure. Two different input populations were recombined; the blue squares are all repaired models (R); the red dots represent the un-repaired model ensembles (NR). The green squares are the recombined results for repaired (R GA) and the orange dots are the refined results for un-repaired protein models (NR GA).

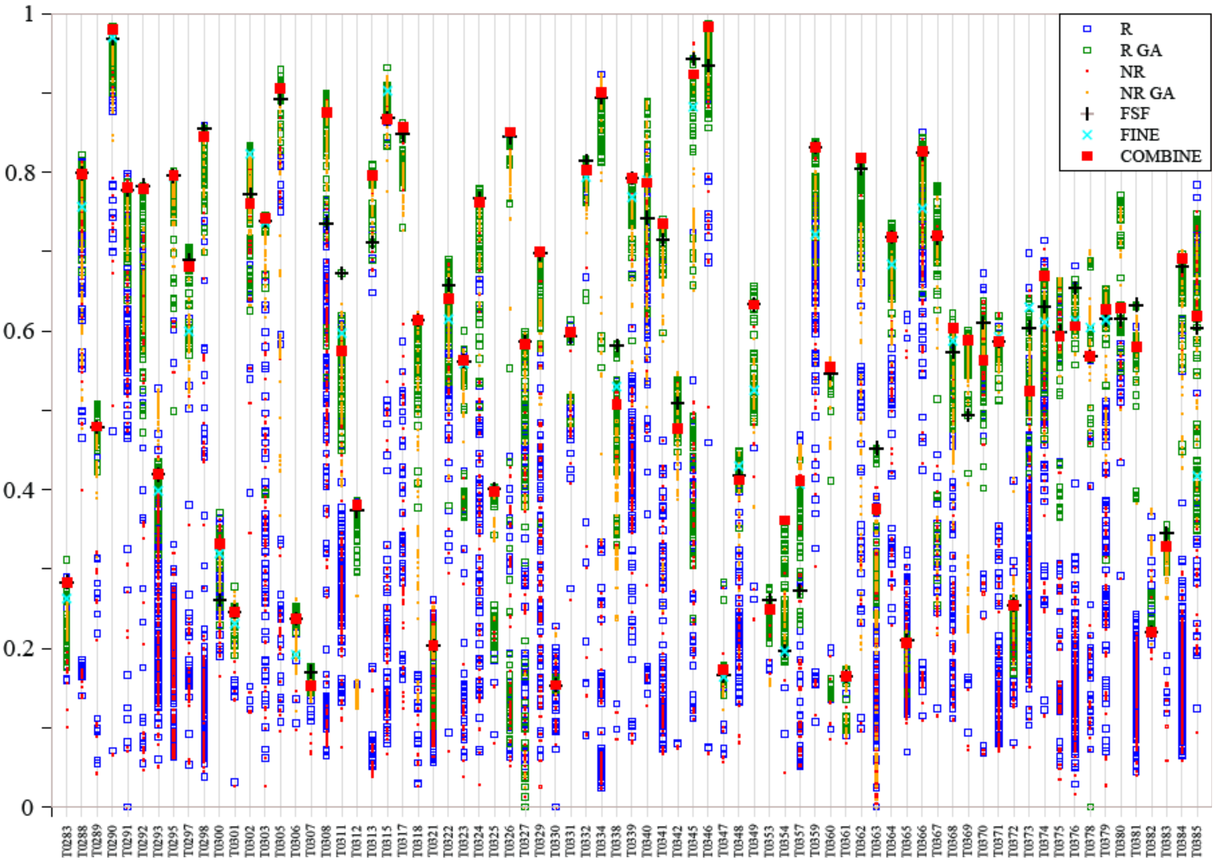

Figure S3 Energy driven GA runs.

For all 75 targets the input populations and final populations after refinement, are shown. The y-axis shows the SC score to the native protein structure. Two different input populations were recombined; the blue squares are all repaired models (R); the red dots represent the un-repaired model ensembles (NR). The green squares are the recombined results for repaired (R GA) and the orange dots are the recombined results for un-repaired protein models (NR GA). The red square is the model selected by the combined, weighted, sequence-identity dependant score. The model selected using the FSF is indicates by a plus symbol, the model selected with the fine energy score is a cyan cross.

Server

The modelling pipeline is integrated into the new server 3D-JIGSAW v3.0. The user can create models for a chosen protein sequence in an automatic and an interactive mode. Furthermore an upload mode is offered where the user can upload their own models for recombination (see supplementary Figure S4).

For all modes, the five best models are available for download or direct inspection. The list of used templates, including their function, is returned. The top alignment is given as well as the sequence predictions such as PSIPRED or DISOPRED[7]. A plot

of the energy profile during the GA protocol is included. For each model the coverage, the combined energy and a Ramachandran plot can be displayed (see supplementary Figure S5).

In the interactive mode an intermediate results page is created, where the indentified templates are visualised, including the function, sequence identity and coverage (see supplementary Figure S6). Each alignment can be inspected and manually adapted. Single models can be selected for model building without recombination. Alternatively, several models can be selected for recombination.

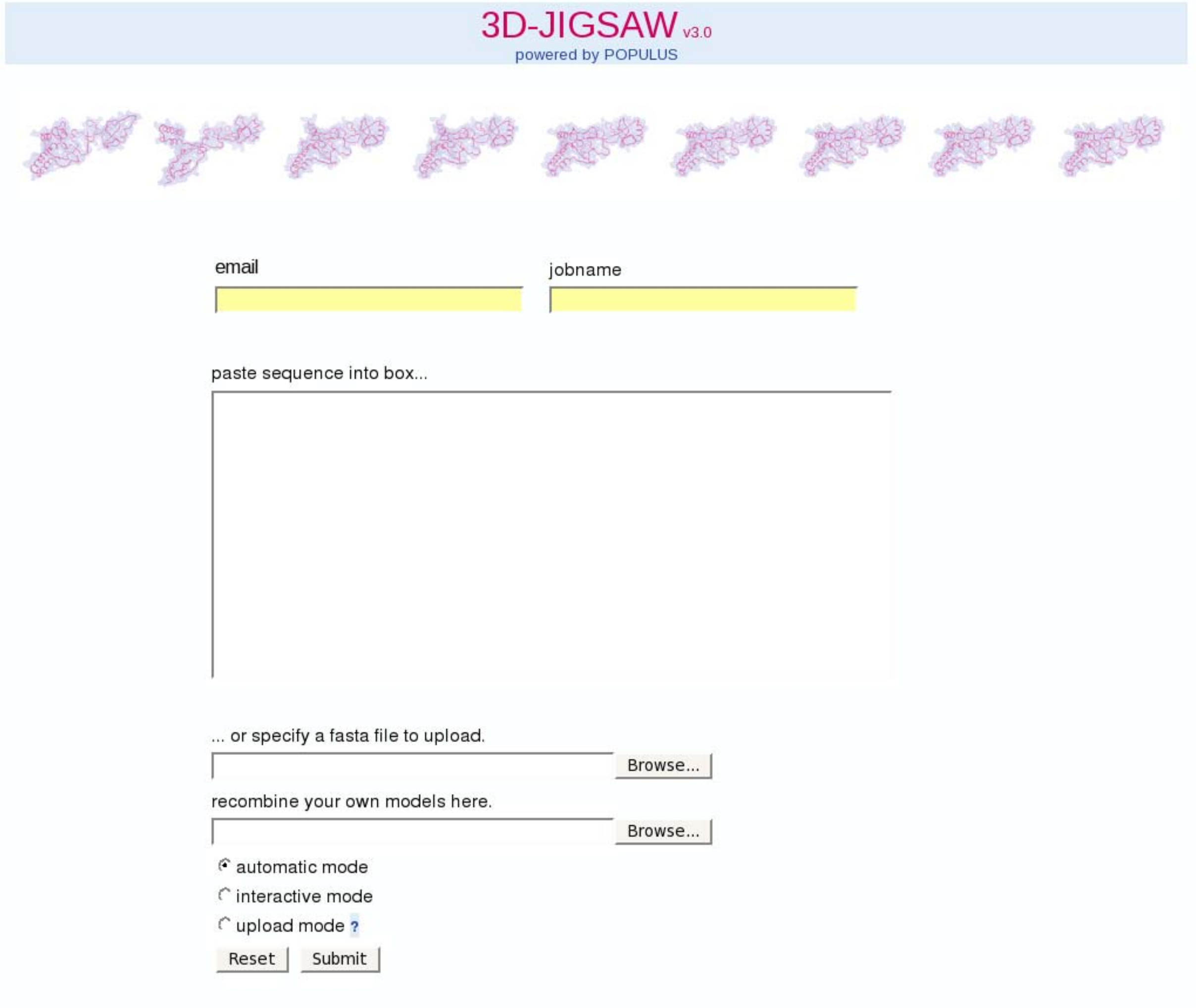

Figure S4 Input mask 3D-JIGSAW v3.0 server.

The user can choose between three different modes: automatic, interactive and upload mode. For each mode either the sequence to be modelled needs to be pasted into the text field, or a sequence file in FASTA format needs to be defined for upload. For the upload mode the user needs to define a file including all the models to be recombined, separated by TER tags.

Sequence predictions:

```
           __________10__________20__________30__________40__________50__________60__________70__________80__________9
AA:        MDELELRIRKAEKLVQDAKKEFEMGLYERCCSTAYYAMFHAAKAMLLGYGRDSKTHRGTIYLIWECREELGLSDDDCSKLSRAFDLREES
Pred:      CCHHHHHHHHHHHHHHHHHHHHHCCCHHHHHHHHHHHHHHHHHHHHHHCCCCCCCCCHHHHHHHHHHHCCCCHHHHHHHHHHHHHHHHC
Conf:      9178899999899999999998617982899999999999999999999806887645689999998761466589999998988732001
Disorder:  DDOOOOOOOOOOOOOOOOOOOOOOOOOOOOOOOOOOOOOOOOOOOOOOOOOOOOOOOOOOOOOOOOOOOOOOOOOOOOOOOOOOOOOOOOO
```

Download sequence prediction files:

PSIPRED secondary structure prediction.

DISOPRED disordered prediction.

POPULUS energy plot:

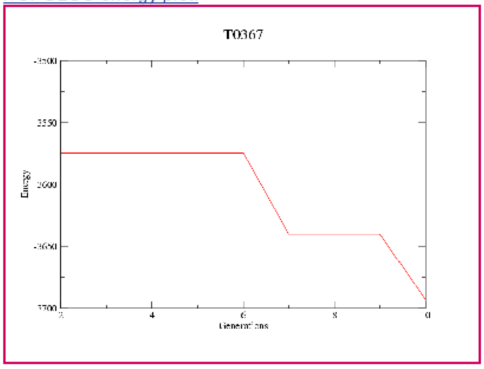

Download models:

MODEL	ENERGY	COVERAGE	START	END	SEQ COVERAGE	RAMACHANDRAN PLOT	PDB	JMOL
MODEL_1	-7336.65	1.00	1	125			download	view
MODEL_2	-7282.81	1.00	1	125			download	view

Figure S5 Final results page.

For the final results sequences predictions, the POPULUS energy profile and up to five different models are given. For each model an energy score, the coverage and a Ramachandran Plot can be displayed.

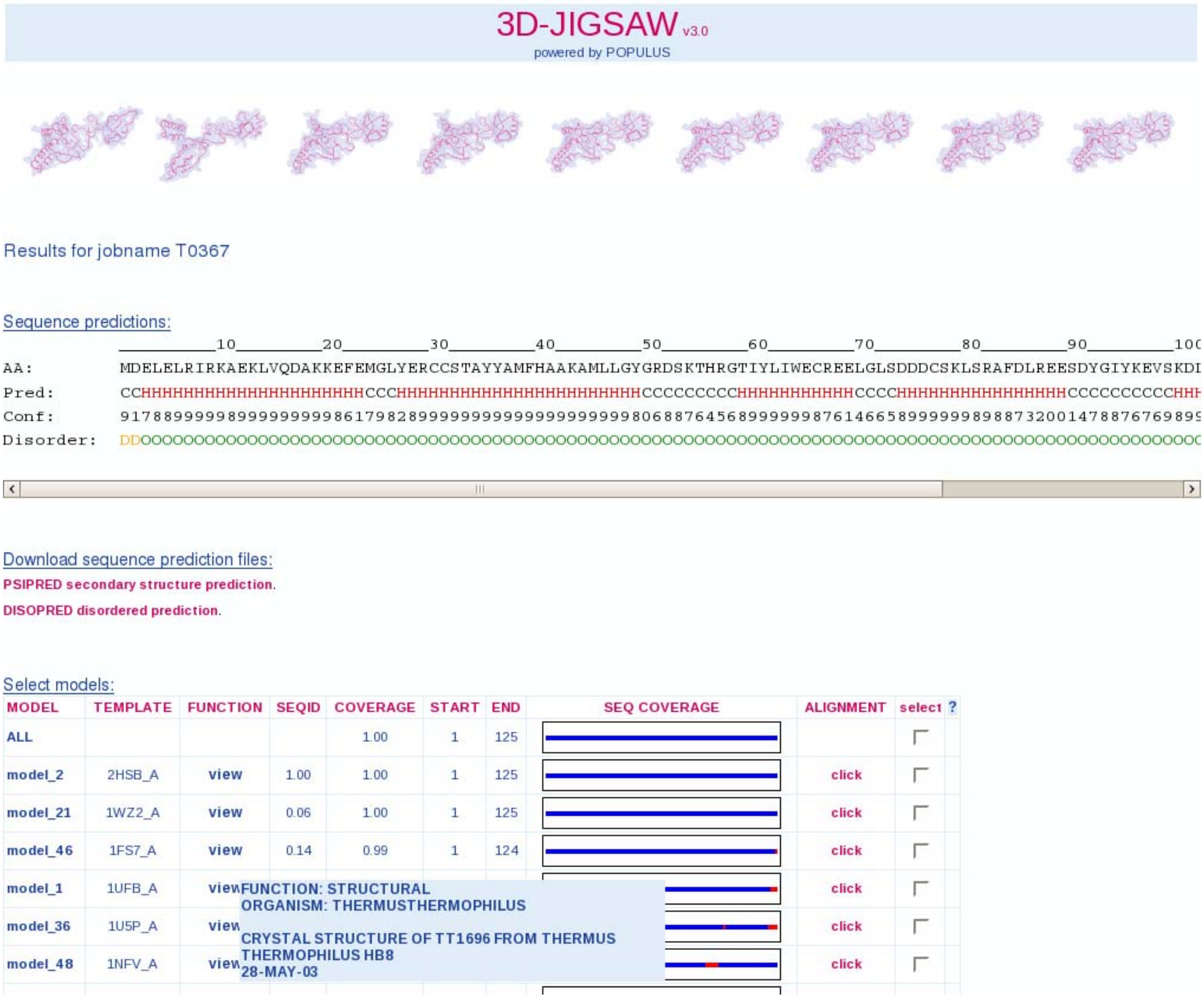

Figure S6 Interactive mode results page.

In the interactive mode an intermediate results page is created. Here, the different sequence predictions are available. For each model, the template, the template function, the sequence identity and the sequence coverage is given. The alignments can be adapted manually and models can either be selected for single modelling or for recombination.

References

1. Canutescu AA, Dunbrack RL, Jr.: **Cyclic coordinate descent: A robotics algorithm for protein loop closure**. *Protein Sci* 2003, **12**(5):963-972.
2. Fitzkee NC, Fleming PJ, Rose GD: **The Protein Coil Library: a structural database of nonhelix, nonstrand fragments derived from the PDB**. *Proteins* 2005, **58**(4):852-854.
3. Ramachandran GN, Sasisekharan V: **Conformation of polypeptides and proteins**. *Adv Protein Chem* 1968, **23**:283-438.
4. Chenna R, Sugawara H, Koike T, Lopez R, Gibson TJ, Higgins DG, Thompson JD: **Multiple sequence alignment with the Clustal series of programs**. *Nucleic Acids Res* 2003, **31**(13):3497-3500.
5. Jones DT: **Protein secondary structure prediction based on position-specific scoring matrices**. *J Mol Biol* 1999, **292**(2):195-202.
6. Offman MN, Fitzjohn PW, Bates PA: **Developing a move-set for protein model refinement**. *Bioinformatics* 2006, **22**(15):1838-1845.
7. Ward JJ, Sodhi JS, McGuffin LJ, Buxton BF, Jones DT: **Prediction and functional analysis of native disorder in proteins from the three kingdoms of life**. *J Mol Biol* 2004, **337**(3):635-645.
